# Hand-assisted laparoscopic surgery versus laparoscopic right colectomy: a meta-analysis

**DOI:** 10.1186/s12957-017-1277-2

**Published:** 2017-12-04

**Authors:** Guosen Wang, Jianping Zhou, Weiwei Sheng, Ming Dong

**Affiliations:** Department of Gastrointestinal Surgery & Hernia and Abdominal Wall Surgery, The First Hospital, China Medical University, Shenyang, Liaoning Province China

**Keywords:** Hand-assisted laparoscopic surgery, Laparoscopic right colectomy, Meta-analysis

## Abstract

**Objective:**

The objective of this study is to systematically assess the clinical efficacy of hand-assisted laparoscopic surgery (HALS) and laparoscopic right colectomy (LRC).

**Methods:**

The randomized controlled trials (RCTs) and non-RCTs were collected by searching electronic databases (Pubmed, Embase, and the Cochrane Library). The outcomes included intraoperative outcomes, postoperative outcomes, postoperative morbidity, and oncologic outcomes. Meta-analysis was performed using of RevMan 5.3 software.

**Results:**

A total of five studies involving 438 patients were finally included, with 202 cases in HALS group and 236 cases in LRC group. Results of meta-analysis showed that there was no statistical difference between HALS and LRC in terms of conversion rate, length of hospital stay, reoperation rate, postoperative morbidity, and oncologic outcomes. The operative time was 6.5 min shorter in HALS group; however, it was not a clinically significant difference. Although the incision length was longer in HALS, it did not influence the postoperative recovery.

**Conclusions:**

HALS can be considered an alternative to LRC which combines the advantages of open as well as laparoscopic surgery.

## Background

Since the first report of laparoscopic colectomy by Jacobs et al. [1] in the early 1990s, laparoscopic surgery has been established in the treatment of colorectal diseases. Laparoscopic colorectal surgery could achieve similar oncological outcomes compared with open surgery [2, 3], and it has certain potential advantages, include less blood loss, faster postoperative recovery, less pain, and less wound-related complications [4–7]. Although its benefits have been well documented, laparoscopic right colectomy (LRC) has still not been widely used [8, 9]. This is probably due to the fact that the procedure is technically difficult, lacking of tactile feedback, and time-consuming and needs sufficient operative volume to ascend the learning curve [10–12].

Hand-assisted laparoscopic surgery (HALS) was introduced to simplify the procedure while preserving the clinical advantages of minimally invasive surgery [13]. Comparing with LRC, HALS decreases the learning curve by restoring tactile feedback, and the intracorporeal hand can be used to blunt dissection, retraction, and rapid control of bleeding. On the other hand, HALS can result in a larger incision and a more invasive procedure and interfere with the field of vision [14].

To date, it is controversial as to whether HALS or LRC is preferred in the treatment of colorectal diseases. Proponents believe HALS facilitates minimally invasive colorectal resection for patients with complicated situation, such as obese patients and individuals with a bulky tumor. Critics believe that HALS discourages the use of laparoscopic methods and results in notably longer incisions.

There are several studies comparing HALS and LRC, but no single study provides evidence which procedure is better. Therefore, a meta-analysis is carried out comparing HALS and LRC.

## Methods

The review was performed based on the PRISM (Preferred Reporting Items for Systematic Reviews and Meta-analyses) statement [15].

### Study search

Electronic databases (Pubmed, Embase, and the Cochrane Library) were searched without limits of date and language. The search terms were as follows: (“hand-assisted laparoscopic” OR “HALS”) AND (“laparoscopic” OR “laparoscopic-assisted” OR “conventional laparoscopy”) AND (“right hemicolectomy” OR “right colectomy”). Additionally, the reference of included studies and related reviews were searched for potentially eligible trials. The search was performed on January 15, 2017.

### Inclusion and exclusion criteria

Inclusion criteria are as follows: (1) patients undergoing right colectomy; (2) clinical studies that compared HALS versus LRC on surgical outcomes; (3) be randomized controlled trials (RCTs) or non-RCTs, no language restrictions; (4) studies were required to report on at least one of the outcomes of interest as following: intraoperative outcomes, postoperative outcomes, postoperative morbidity and oncologic outcomes.

Exclusion criteria are as follows: (1) full-test cannot be obtained and (2) no outcome data of interest.

### Study selection

Two reviewers independently selected studies according to the inclusion and exclusion criteria, and disagreement was resolved through discussion. The EndNote X7 software was used to remove duplicate records, then the titles and abstracts were screened to identify potential articles, and full texts were retrieved and reviewed to decide whether the studies were eligible.

### Data extraction

Two reviewers independently extracted the following data into preformatted table: (1) Study characteristics: first author, year of publication, study design, country, and histologic diagnosis; (2) Baseline data of patients: number of cases, mean age, sex ratio, body mass index (BMI), and American Society of Anesthesiologists (ASA) score; (3) Intraoperative outcomes: operative time, incision length, and conversion rate; (4) Postoperative outcomes: length of hospital stay and reoperation rate; (5) Postoperative morbidity: postoperative overall complications, wound infection, anastomotic leak, and ileus; (6) Oncologic outcomes: number of harvested lymph node, recurrence rate, and death rate.

### Quality assessment

The quality of included RCTs was assessed by using the Cochrane Collaboration’s risk of bias tool [16], including the following domains: selection bias, performance bias, detection bias, attrition bias, reporting bias, and other bias. The quality of included non-RCTs was assessed by using the Newcastle–Ottawa Scale (NOS) [17], including three main items: patient selection, comparability, and exposure of HALS and LRC groups. The scale used a star system, which ranged from 0 to 9 stars, and studies were considered as high quality if achieving 7 stars or more. The assessments were performed by two reviewers separately, with inconsistency resolved by discussion.

### Statistical analysis

The meta-analysis was performed using RevMan 5.3 software. For categorical variables or continuous variables, odds ratios (ORs) or weighted mean differences (WMDs) with the corresponding 95% confidence interval (CI) were used as the summary statistic respectively, and *P* < 0.05 was considered statistically significant. If the studies reported continuous variables as medians with ranges, we assumed that the mean is equal to the medians and estimated the standard deviation (SD) as range/4 (samples ≤ 70) or range/6 (samples > 70) [18]. The homogeneity among the included studies was assessed using the *I* ^2^ statistic and the *x* ^2^ test. The fixed effects model was performed for studies with low heterogeneity (*I* ^2^ ≤ 50% and *P* > 0.05). In the presence of significant heterogeneity (*I* ^2^ > 50% or *P* < 0.05), the random effect model was used to pool the data. The pooled results were expressed by forest plots, and funnel plot was used to estimate for publication bias from a fixed effects model.

## Results

### Studies selection

**Fig. 1:**
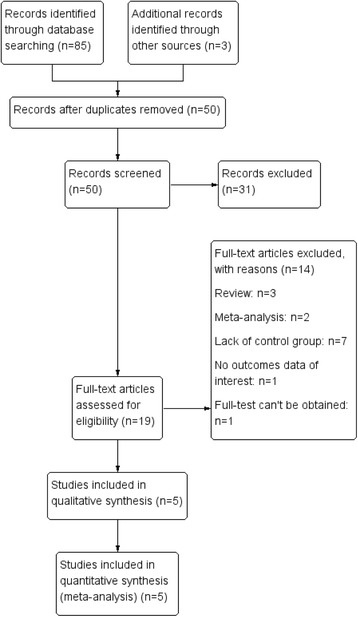
Systematic search and study selection

A total of 88 studies were retrieved by the primary search, and five clinical studies were eligible for the meta-analysis eventually [19–23], involving 438 patients of whom 202 in HALS group and 236 in LRC group. Figure 1 shows the detailed process of the studies selection. Among the included trials, three trials [19, 20, 22] compared malignant cases between HALS and LRC, and two trials [21, 23] compared malignant and benign cases, whose sample size ranged from 58 to 127. The study characteristics and baseline data of patients are listed in Table 1.

**Table 1:** Study characteristics and baseline data of patients

Study	Study characteristics			Baseline data of patients				
	Design	Country	Histologic diagnosis	No. of patients	Age (years)	Sex (M/Fe)	BMI (kg/m^2^)	ASA score (1/2/3/4)
Bae et al. [19]	Non-RCT	Korea	Cancer	HALS 53	68 (30–85)	34/19	22.9 (16.2–32.9)	18/23/10/2
				LRC 45	63 (36–90)	19/26	23.6 (18.7–32.3)	16/23/6/0
Ng et al. [20]	RCT	China	Cancer	HALS 30	73.5 (34–85)	9/21	21 (15.3–26.6)	NA
				LRC 30	70.8 (34–89)	10/20	21 (15.3–34.7)	NA
Papaconstantinou et al. [21]	Non-RCT	USA	Cancer, Polyp, Crohn	HALS 29	60.3 ± 13.5	13/16	30.3 ± 6.2	0 14/15/0
				LRC 29	61.1 ± 11.6	13/16	28.5 ± 5.3	1/14/13/1
Qiu et al. [22]	Non-RCT	China	Cancer	HALS 47	60.6 ± 12.4	22/25	NA	NA
				LRC 48	63.1 ± 13.1	25/23	NA	NA
Vogel et al. [23]	Non-RCT	USA	Cancer, Polyp, Crohn, Other	HALS 43	67 ± 13	27/16	28.4 ± 6.7	2/17/23/1
				LRC 84	66 ± 14	44/40	28.6 ± 6.5	1/33/43/7

*HALS* hand-assisted laparoscopic surgery, *LRC* laparoscopic right colectomy, *RCT* randomized controlled trial, *No* number, *M/Fe* male/female, *BMI* body mass index, *ASA* American Society of Anesthesiologists, *NA* not available

### Quality judgments of studies

The RCT [20] was of high risk of bias, and the four non-RCTs [19, 21–23] achieved NOS score of 7 or more, which showed the included studies were high quality. The quality assessment is detailed in Table 2.

**Table 2:** Quality assessment of included studies using the Cochrane Collaboration’s Risk of Bias Tool and the Newcastle–Ottawa Scale (NOS)

Design	Study	Selection bias	Performance bias			Detection bias	Attrition bias	Reporting bias		Other bias
RCT	Ng et al. [20]	Low risk	High risk			High risk	Low risk	Low risk		Unclear risk
Design	Study	Selection				Comparability	Exposure			Quality judgment
		1	2	3	4	5	6	7	8	
non-RCTs	Bae et al. [19]	★	★		★	★	★	★	★	7
	Papaconstantinou et al. [21]	★	★		★	★★	★	★	★	8
	Qiu et al. [22]	★	★		★	★★	★	★	★	8
	Vogel et al. [23]	★	★		★	★	★	★	★	7

Selection: 1. Is the case definition adequate? 2. Representativeness of the cases; 3. Selection of controls; 4. Definition of controls. Comparability: 5. Did the study have no differences between hand-assisted laparoscopic surgery and laparoscopic right colectomy? Five main factors were considerate: histologic diagnosis, age, sex, BMI, and ASA. Exposure: 6. Ascertainment of exposure; 7. Same method of ascertainment for cases and controls; 8. Non-response rate

★ It stands for one score in the assessment of study quality

★★ It stands for two scores in the assessment of study quality

### Operative time

**Fig. 2:**
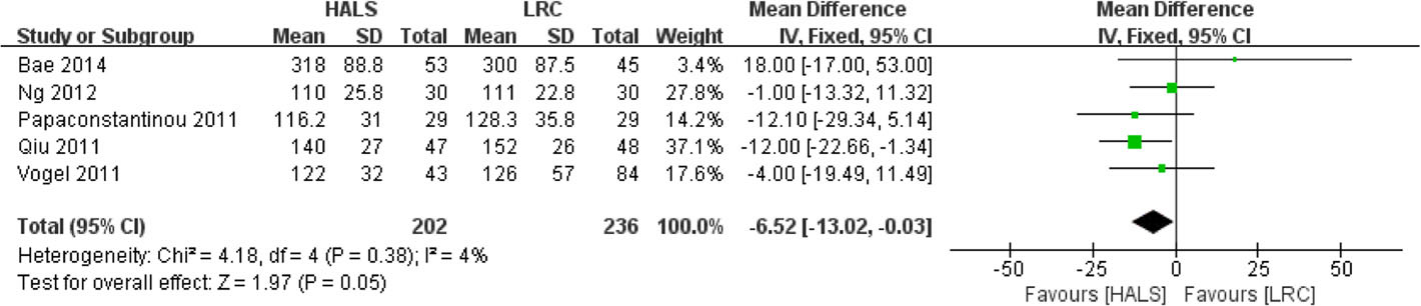
Forest plot of operative time comparing HALS and LRC

All five studies involving 438 patients reported available data on the operative time, and low heterogeneity was observed among the trails (*I* ^2^ = 4%, *P* = 0.38). Meta-analysis result showed that operative time was 6.5 min shorter in HALS compared with LRC (WMD = − 6.52; 95% CI − 13.02, −  0.03; *P* = 0.05; Fig. 2). However, it was not a clinically significant difference.

### Incision length

**Fig. 3:**
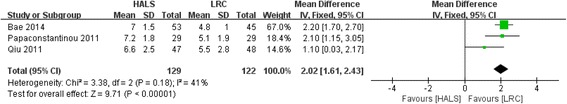
Forest plot of incision length comparing HALS and LRC

Three studies involving 251 patients reported available data on the incision length, and low heterogeneity was observed among the trails (*I* ^2^ = 41%, *P* = 0.18). Meta-analysis result showed that HALS was associated with longer incision length compared with LRC (WMD = 2.02; 95% CI 1.61, 2.43; *P* < 0.0001; Fig. 3).

### Conversion rate

**Fig. 4:**
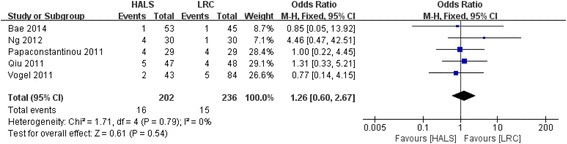
Forest plot of conversion rate comparing HALS and LRC

All five studies involving 438 patients reported available data on the conversion rate, and low heterogeneity was observed among the trails (*I* ^2^ = 0%, *P* = 0.79). Meta-analysis result showed no significant difference between HALS and LRC in conversion rate (OR = 1.26; 95% CI 0.60, 2.67; *P* = 0.54; Fig. 4).

### Length of hospital stay

**Fig. 5:**
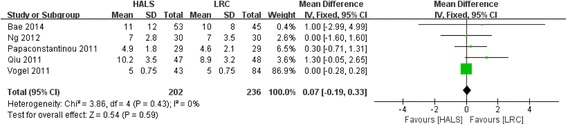
Forest plot of length of hospital stay comparing HALS and LRC

All five studies involving 438 patients reported available data on the length of hospital stay, and low heterogeneity was observed among the trails (*I* ^2^ = 0%, *P* = 0.43). Meta-analysis result showed no significant difference between HALS and LRC in length of hospital stay (WMD = 0.07; 95% CI − 0.19, 0.33; *P* = 0.59; Fig. 5).

### Reoperation rate

**Fig. 6:**
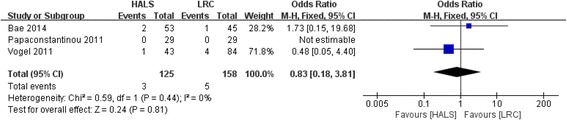
Forest plot of reoperation rate comparing HALS and LRC

Three studies involving 283 patients reported available data on the reoperation rate, and low heterogeneity was observed among the trails (*I* ^2^ = 0%, *P* = 0.44). Meta-analysis result showed no significant difference between HALS and LRC in reoperation rate (OR = 0.83; 95% CI 0.18, 3.81; *P* = 0.81; Fig. 6).

### Postoperative overall complications

**Fig. 7:**
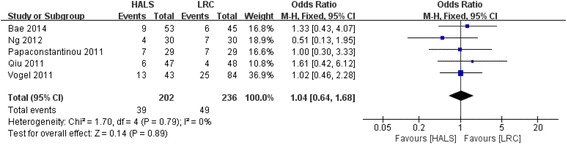
Forest plot of postoperative overall complications comparing HALS and LRC

All five studies involving 438 patients reported available data on the postoperative overall complications, and low heterogeneity was observed among the trails (*I* ^2^ = 0%, *P* = 0.79). Meta-analysis result showed no significant difference between HALS and LRC in postoperative overall complications (OR = 1.04; 95% CI 0.64, 1.68; *P* = 0.89; Fig. 7).

### Wound infection

**Fig. 8:**
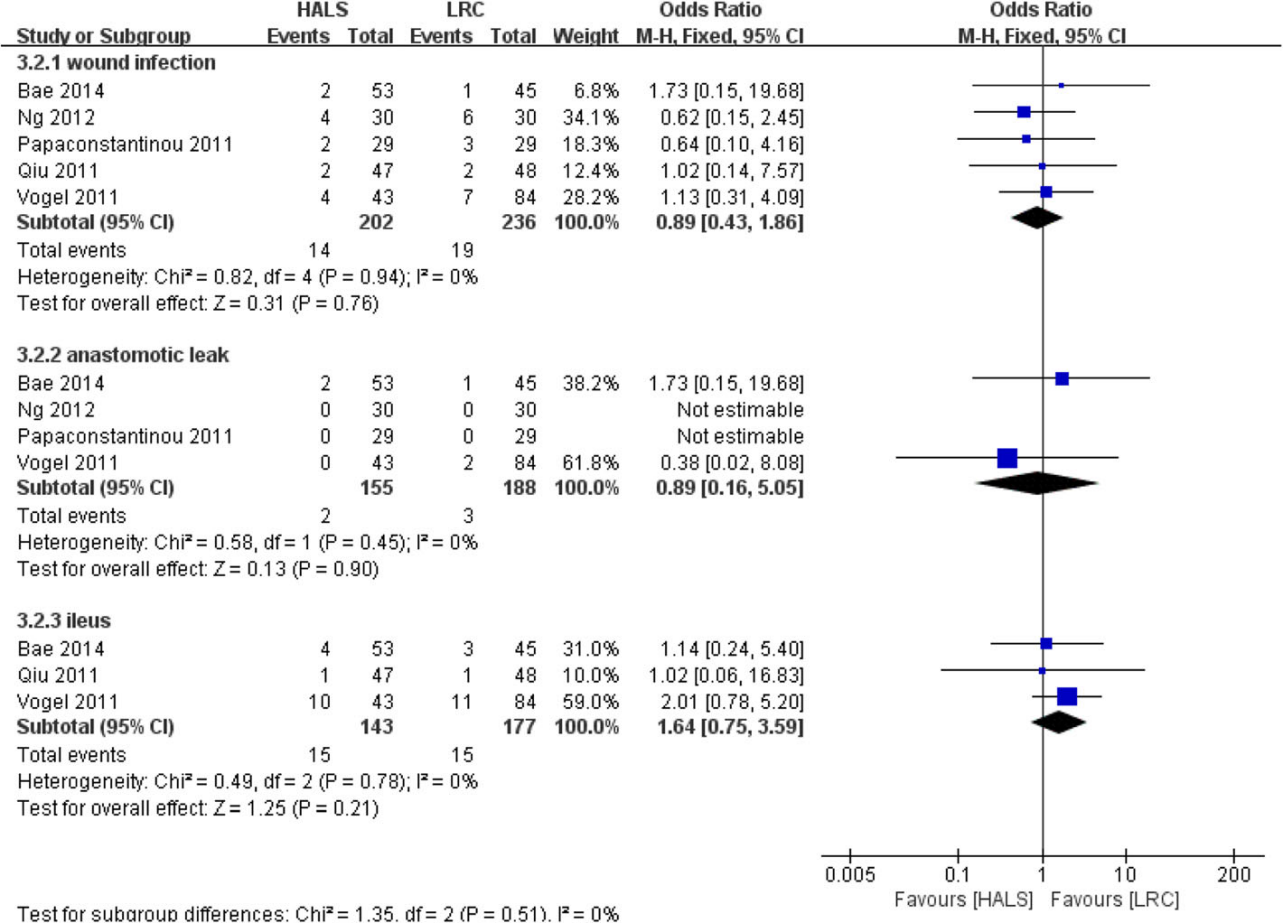
Forest plot of wound infection, anastomotic leak, and ileus comparing HALS and LRC

All five studies involving 438 patients reported available data on the wound infection, and low heterogeneity was observed among the trails (*I* ^2^ = 0%, *P* = 0.94). Meta-analysis result showed no significant difference between HALS and LRC in wound infection (OR = 0.89; 95% CI 0.43, 1.86; *P* = 0.76; Fig. 8).

### Anastomotic leak

Four studies involving 343 patients reported available data on the anastomotic leak, and low heterogeneity was observed among the trails (*I* ^2^ = 0%, *P* = 0.45). Meta-analysis result showed no significant difference between HALS and LRC in anastomotic leak (OR = 0.89; 95% CI 0.16, 5.05; *P* = 0.90; Fig. 8).

### Ileus

Three studies involving 320 patients reported available data on the ileus, and low heterogeneity was observed among the trails (*I* ^2^ = 0%, *P* = 0.78). Meta-analysis result showed no significant difference between HALS and LRC in ileus (OR = 1.64; 95% CI 0.75, 3.59; *P* = 0.21; Fig. 8).

### Number of lymph node harvested

**Fig. 9:**
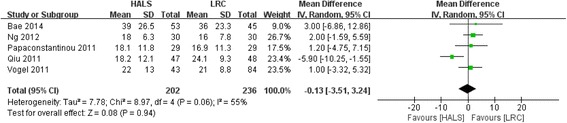
Forest plot of number of lymph node harvested comparing HALS and LRC

All five studies involving 438 patients reported available data on the number of lymph node harvested, and significant heterogeneity was observed among the trails (*I* ^2^ = 55%, *P* = 0.06). Meta-analysis result showed no significant difference between HALS and LRC in number of lymph node harvested (WMD = − 0.13; 95% CI − 3.51, 3.24; *P* = 0.94; Fig. 9).

### Recurrence and death rate

**Fig. 10:**
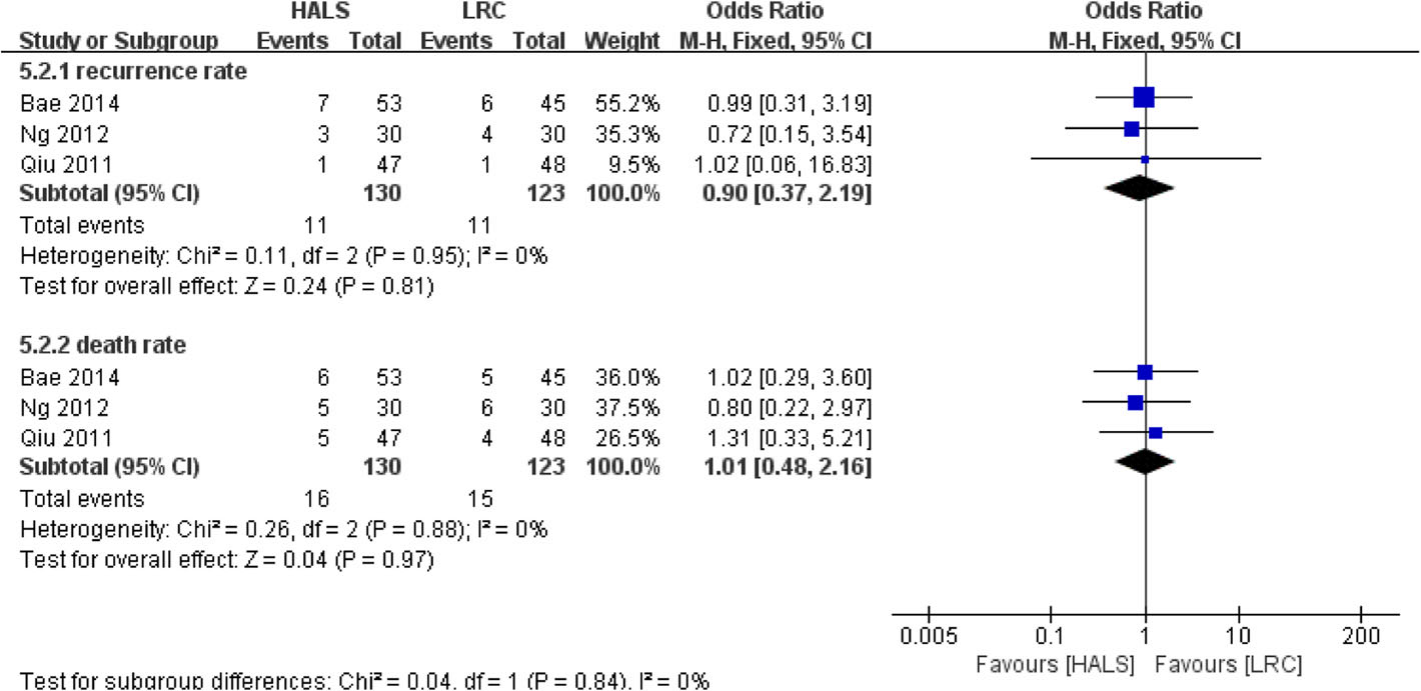
Forest plot of recurrence and death rate and comparing HALS and LRC

Three studies involving 253 patients reported available data on the recurrence and death rate, and low heterogeneity was observed among the trails (*I* ^2^ = 0%, *P* > 0.88). Meta-analysis result showed no significant difference between HALS and LRC in recurrence rate (OR = 0.90; 95% CI 0.37, 2.19; *P* = 0.81; Fig. 10) and death rate (OR = 1.01; 95% CI 0.48, 2.16; *P* = 0.97; Fig. 10).

### Assessment of publication bias

**Fig. 11:**
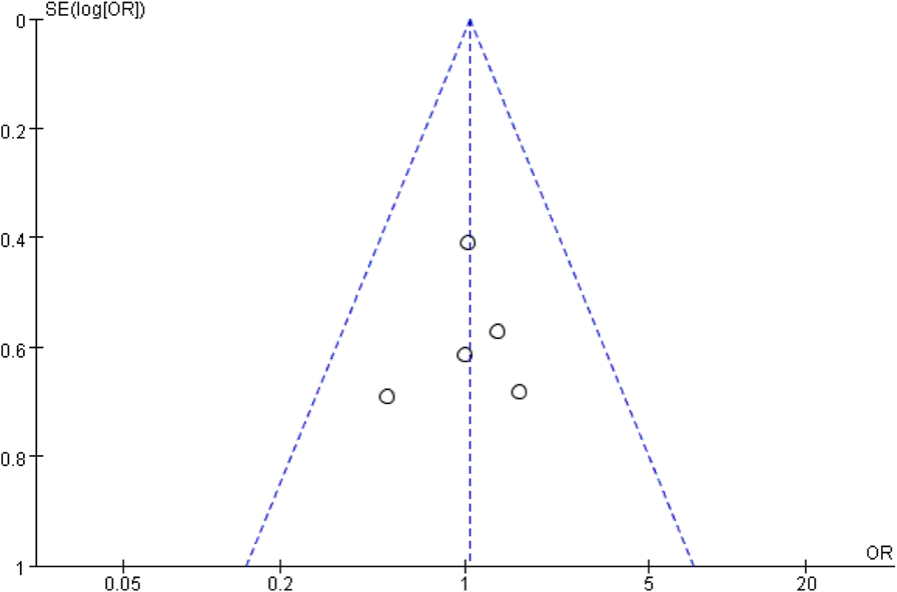
Funnel plot of postoperative overall complications

Funnel plot analysis was performed on the postoperative overall complications. As showed, there was low risk of publication bias in our meta-analysis (Fig. 11).

## Discussion

Jacobs et al. [1] reported the first laparoscopic colectomy in the early 1990s. Comparing with open surgery, laparoscopic colorectal surgery has certain potential advantages [4–7], as well as similar oncological outcomes [2, 3]. HALS is a hybrid procedure which allows the surgeon to insert the non-dominant hand into the abdomen through a special hand-access device while still maintaining the pneumoperitoneum [24]. Comparing with LRC, HALS decreases the learning curve by restoring tactile feedback, and the intracorporeal hand can be used to blunt dissection, retraction, and rapid control of bleeding. It is controversial as to whether HALS or LRC is preferred at present. There were two previously published systematic reviews on the subject comparing HALS and laparoscopic approach in colorectal surgery [25, 26], rather than comparing HALS and LRC. Therefore, a meta-analysis is carried out.

The meta-analysis that compares HALS and LRC showed that there was no statistical difference between HALS and LRC in terms of conversion rate, length of hospital stay, reoperation rate, postoperative morbidity, and oncologic outcomes. The operative time was 6.5 min shorter in HALS group; however, it was not a clinically significant difference. Although the incision length was longer in HALS, it did not influence the postoperative recovery.

The one previously published systematic review showed the operative time was shorter in HALS comparing with laparoscopic approach [25]. Meijer et al. [27] found that “surgical action efficiency” ratio was 0.55 for HALS and 0.71 for laparoscopic colorectal surgery, which indicated HALS was more efficient. Leblanc et al. [28] performed sigmoid colectomy on an augmented reality simulator and showed HALS reduced operative time by accelerating colonic mobilization and anastomosis. In our meta-analysis, the operative time was 6.5 min shorter in HALS group and was the same as Aalbers et al. reported [25]. Pandya et al. [29] reported the most common reasons for conversion were diverticular inflammation, intraabdominal adhesions, and need for distal rectal resection. Biondi et al. [30] showed conversion occurred mostly because of invasion to adjacent structures, bulky tumor, and adhesions, and converted patients had worse survival than laparoscopic completed patients. It was worth noting that laparoscopic colorectal surgery could convert to HALS or laparotomy [31–33], which indicated HALS was more suitable for complicated situation.

The incision length of HALS was longer in this meta-analysis, but it did not influence the postoperative recovery in terms of length of hospital stay, reoperation rate, and postoperative morbidity. Marcello et al. [32] reported a multicenter prospective randomized trial and showed postoperative recovery was similar between HALS and laparoscopic colorectal surgery. Regarding the number of trocars used, HALS is performed using a hand port and two to three trocars. On the other hand, LRC is performed using four to five trocars. The hand port in HALS can restore the tactile sensation and is used as an extraction site. LRC makes an extra small incision to extract the specimen at the end of operation. Both the port site (in HALS) and the extraction site (in LRC) are potential sites for future ventral hernia, and Bae et al. [19] showed there was no statistical difference between HALS and LRC.

Radical surgery of malignant tumor included enough distance of resection margin and number of lymph nodes harvested. There was evidence that positive circumferential margin in rectal cancer was adverse effect of recurrence [34], and Chang et al. [35] reported that survival would increase when a greater lymph node was resected in the patients of II or III colon cancer. Our pooled results showed the oncologic outcomes have no statistical difference between HALS and LRC with respect to number of harvested lymph node, recurrence rate, and death rate.

With regard to cost, Qiu et al. [22] reported the total costs of HALS were less than those of LRC. HALS used conventional staplers rather than expensive laparoscopic staplers, and the trocars were less used in HALS; thus, the total costs could be partly offset in spite of the hand device being expensive. Roslani et al. [36] performed a comprehensive cost analysis and found the total costs were similar between HALS and laparoscopic colorectal surgery.

There were limitations in this meta-analysis. (1) Only one RCT [20] was available to include. Because of the considerable clinical heterogeneity of non-RCTs, the power of pooled results may be declined. (2) The sample size was great difference, and patients came from different countries among the included studies, but the quality of included studies was satisfying. (3) The pooled result of harvested lymph node number existed significant statistical heterogeneity, thus reduced the dependability of evidence.

## Conclusions

In conclusion, comparing with LRC, the incision length was longer in HALS, but it did not influence the postoperative recovery. Although the operative time was 6.5 min shorter in HALS, it was not a clinically significant difference. There was no statistical difference in terms of conversion rate, length of hospital stay, reoperation rate, postoperative morbidity, and oncologic outcomes between HALS and LRC. Therefore, HALS can be considered an alternative to LRC which combines the advantages of open as well as laparoscopic surgery. Additional multicenter randomized controlled trials are necessary to fully compare the clinical outcomes between HALS and laparoscopic procedure for right colectomy.

## Abbreviations

ASA: American Society of Anesthesiologists
BMI: Body mass index
HALS: Hand-assisted laparoscopic surgery
LRC: Laparoscopic right colectomy
M/Fe: Male/female
NA: Not available
No: Number
RCT: Randomized controlled trial
